# Advancing road safety strategy development: A data-driven multi-objective optimisation integrated approach

**DOI:** 10.1016/j.heliyon.2024.e34293

**Published:** 2024-07-09

**Authors:** Bosong Jiao, Harry Evdorides

**Affiliations:** School of Engineering, University of Birmingham, Edgbaston, Birmingham, B15 2TT, United Kingdom

**Keywords:** Road safety, Strategic management, Data-driven, Resource allocation

## Abstract

Road traffic accidents pose a significant global health concern, with an alarming 1.19 million fatalities reported in 2021. Traditionally, strategies to address this challenge have relied on expert input and subjective evaluations. This study introduces an adaptive and novel two-stage model that minimizes expert interference by integrating multi-objective optimisation with association rule mining. This innovative approach provides a systematic framework to enhance the efficiency of decision-making and optimise resource allocation outcomes in road infrastructure management, facilitating adaptability to diverse objectives. A case study in Utrecht from iRAP validates the efficiency of the approach, demonstrating significant improvements across various objectives by using the non-dominant sorting genetic algorithm and enabling road local authorities to tailor investment plans to their specific road network characteristics by crash database mining. However, the methodology requires refinement, particularly in identifying risk levels considering the interactive effects of multiple road attributes. In conclusion, while representing a substantial advancement, further refinement is necessary to fully realize its potential in enhancing road safety.

## 1 Introduction

Road traffic collisions represent a significant global health concern, contributing substantially to both death and disability. As reported by the World Health Organization (2023), an estimated 1.19 million fatalities resulted from road traffic incidents in 2021, with a corresponding fatality rate of 15 deaths per 100,000 individuals. Road traffic injury remains the 12th leading cause of death when all ages are considered. These incidents not only result in immense human suffering but also impose significant economic costs, including medical expenses, lost productivity, and damage to infrastructure. This collective strain poses tough challenges for stakeholders ranging from policymakers and road authorities to the entire society. The core to address this challenge is the efforts to identify underlying problems and implement targeted interventions and countermeasures. Central to this endeavour is the strategic allocation of resources to initiatives aimed at improving road safety outcomes.

Several studies have demonstrated the ability of enhancing road safety through the implementation of structured safety management processes. For instance, the Highway Safety Improvement Program (HSIP) outlined a three-phase approach to allocate resources effectively for road safety, which involves identifying high-risk areas, proposing appropriate countermeasures, and allocating funds within budgetary constraints [1]. This systematic approach ensures an organised assessment of safety priorities and efficient resource allocation.

Similarly, international road assessment programme developed a suite of tools based on iRAP methodology to support road infrastructure safety management. It includes four protocols: risk mapping, star rating, safer road investment plans and performance tracking [2], which aligns with the three-step structure promoted by HSIP. The impressive impact of iRAP is its establishment of a structured framework beginning with section analysis and extending to road network analysis, facilitating the clear visualization of road risk and overall network performance. Concurrently, a systematic procedure is outlined for the identification of countermeasures, leveraging insights gained from risk mapping and star rating assessments.

In 2020, Waka Kotahi NZ Transport Agency [3] developed a definition of ‘one network framework’(ONF) to put people, place and movement at the heart of planning and investment, which allows land use and transport planning to be integrated. The ONF aligns with strategic transport planning at all levels, where road to zero strategy is one of the objectives to achieve zero death on road. Meanwhile, they adopted an investment method that utilized three investment criteria—— government policy statement (GPS) on land transport alignment, scheduling, and efficiency—to prioritize proposed activities[4]. It is an interesting and decisive transport funding allocation framework aligning with strategic objectives.

In recent years, scholars and practitioners have investigated diverse methodologies and frameworks aimed at optimising the allocation of resources for road safety. These approaches involve traditional cost-benefit analyses to prioritize countermeasures, as well as more advanced optimisation techniques for selecting combinations of interventions. However, the search for an optimal investment plan in road safety strategic management is hindered by a variety of practical constraints. These constraints often conflict, leading to trade-offs between benefits, efficiency, and costs. Additionally, the unmeasurable impacts on road network operations exacerbate these challenges. These conflicting objectives and unaccountable effects emphasize the complexities and gaps in current road safety strategic management practices. Decision-making subjectivity and expert preferences often overlook those pertinent road network issues, highlighting the need for advanced and comprehensive optimisation methodologies that accommodate diverse objectives with less human intervention.

Given advancements in technology, data analytics, and computation, there is an increasing demand for data-driven methods in optimising road safety measures. Therefore, this study introduces a novel two-stage model that minimizes expert interference by integrating multi-objective optimisation with association rule mining. This innovative approach offers a systematic framework to enhance decision-making efficiency and optimise resource allocation in road infrastructure management. A case study in Utrecht, validated by iRAP, demonstrates the effectiveness of proposed model, achieving significant improvements across various objectives using the non-dominated sorting genetic algorithm. By mining crash databases, the model enables local road authorities to tailor investment plans to their specific network characteristics. While the methodology represents a substantial advancement in reducing reliance on expert judgment, further refinement is needed to account for the interactive effects of multiple road attributes in identifying risk levels.

This study exclusively examines investment planning for road safety, focusing on the third step of the Highway Safety Improvement Program (HSIP). The identification of high-risk areas and the proposal of countermeasures will not be addressed within the scope of this investigation. The remainder of this paper is organized as follows: Section 2 provides an overview of the optimisation related works developed for road safety improvement regarding resource allocation. Section 3 presents the methodology employed in this study, consisting of objectives identification, data collection, a brief introduction of definitions in multi-objective optimisation and integration of analytical techniques. Section 4 presents the findings of optimisation model and the result of the overall methodology based on information of Utrecht safer road investment plan, followed by a discussion in Section 6 to demonstrate the advancement and implications of the proposed methodology for decision making in practice. Finally, Section 6 offers concluding remarks and suggests directions for future research in the field of road safety strategic management.

## 2 Related work

The optimisation of road safety resource allocation is a critical endeavour in road infrastructure engineering, where the aim is to minimise crash or severity for improving overall safety outcomes within constrained budgets. Over the years, researchers have proposed various methodologies and frameworks to address this complex challenge.

The mixed integer linear programming (MILP) model was developed by Banihashemi and Dimaiuta [5] to maximize the benefits obtained by predefined alternatives for different segments of the highway which was achieved by minimizing the expected number of crashes. Their subsequent work [6] extended this model to incorporate travel time considerations, seeking to minimise both crash and delay costs within budget constraints.

Chowdhury et al. [7] introduced a multi-objective approach that emphasizes keeps the objectives in their respective units and provides a set of solutions rather than a single optimal. The core to their methodology is the introduction of disutility values, which estimated the relative severity of different crash levels. By identifying causal factors on road segments and suggesting appropriate countermeasures, the study aims to optimise resource allocation while minimizing expected loss of disutility at all road types.

Lambert et al. [8] proposed a multi-objective framework tailored for guardrail resource allocation, which facilitated interpretation of variety of benefits and costs in their own units. Their approach allows for subjective evaluations of different objectives, accommodating the varied needs and preferences of stakeholders.

Importance segregated multi-objective optimisation (ISMO) model was proposed by Yu et al. [9]. They separated objectives into two levels: decision and supporting level, so that fewer optimal solutions would be obtained from decision level and the process avoided overrating less important objectives.

Mishra et al. [10] presented optimal multiple resource allocation strategies to improve urban intersections under policy and budget constraints. focused on optimal resource allocation strategies for urban intersections, with a particular emphasis on equity in safety benefits and allocation within counties. Their approach considers policy and budget constraints to maximize safety benefits while ensuring fair distribution within communities.

Augeri et al. [11] proposed an interactive multi-objectives optimisation method using dominance-based rough set approach (DRSA) to provide an explicit structure for road safety decision makers. They applied DRSA to formulate decision rules after acquirement of pareto solutions, iteratively refining constraints until an optimal solution is reached.

In multi-objective optimisation, the preferences of the designer regarding the prioritization of objectives are captured [12]. The NSGA-II method is a widely used, fast, and elitist approach for deriving Pareto-optimal solutions across various domains.

Hojjati et al. [13]applied NSGA-II to optimise the operations of two reservoirs in the Ozan River catchment, aiming to maximize income from power generation and enhance flood control capacity. Their comparison with multi-objective particle swarm optimisation (MOPSO) revealed that NSGA-II performed better in optimising the reservoir system, offering improved coverage of the true Pareto front.

Rahimi et al. [14] conducted a comprehensive review and bibliometric analysis of application of NSGA-II in scheduling problems, highlighting its benefits and extensive use across different scheduling contexts. Their review underscored the flexibility and efficiency of NSGA-II in solving complex scheduling issues, emphasizing its adaptability in various scenarios.

Shaygan et al. [15] demonstrated the effectiveness of NSGA-II in spatial multi-objective optimisation for land use allocation, showing how the algorithm balances multiple conflicting objectives. This research illustrated its capability in handling spatial optimisation problems, making it a valuable tool for land use planning.

Ma et al. [16] discussed the concept of multi-objective optimisation and the foundation of NSGA-II, reviewing its family and modifications. They classified NSGA-II applications in engineering into categories such as manufacturing ([17,18]; H. [19]), transportation ([20,21]; C. [22]), supply chain [23,24], among others.

While the literature provides an overview of various methodologies for optimising road safety resource allocation and NSGA-II applications in different industries, there is a notable gap in discussing the integration of emerging data mining technologies with multi-objective optimisation (e.g., NSGA-II) in road safety strategic management. The use of artificial intelligence, machine learning, and big data analytics offers promising opportunities to enhance road safety initiatives. Hence, further exploration of holistic and data-driven optimisation procedures that consider broader socio-economic impacts of road safety interventions is needed.

## 3 Methodology

This section introduces a multi-objective optimisation methodology integrated with association rule mining for road safety resource allocation. The methodology aims to maximize the estimated net present value (NPV) of benefits, minimise the cost per KSI (Killed or Seriously Injured) saved, and account for the service life of countermeasures.

**Fig. 1:**
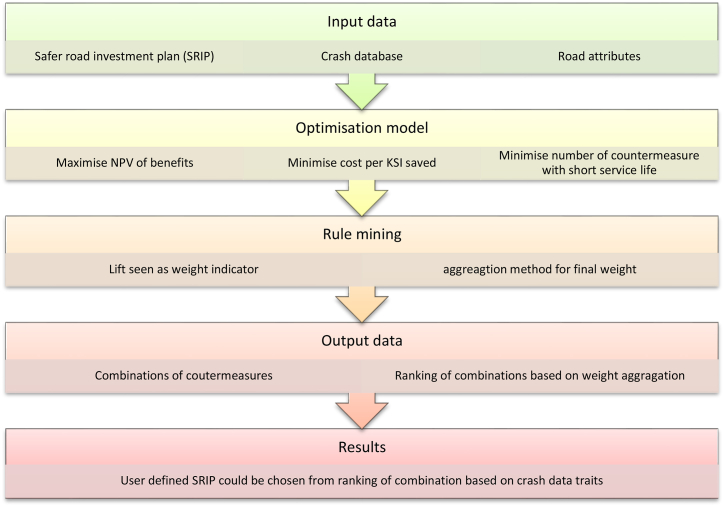
Process of overall methodology.

Since the objectives and the constraints involved are often heterogeneous and conflicting, a multi-objective optimisation methodology is proposed. Decision makers are trying to minimise the overall cost per KSI saved and minimise the construction impact to road network to maximize the overall benefits for road network and reduce KSI crash. This method is a data driven procedure composed of two stages to avoid subjectivity and bias in selection of countermeasures illustrated in Fig. 1. In the first stage, the Pareto optimal set generates a sample of solutions (e.g. Combination of countermeasures) where the non-dominated soring genetic algorithm (NSGA-Ⅱ) was employed. In the second stage, the results of association rule mining are applied to weigh the optimal solutions through aggregation method, where a rule based model expressed in terms of easily understandable “if …, then …” decision rules is used.

### 3.1 Theoretical framework

Obviously, the multi-objective optimisation implies the exploration of optimal solutions, constituting a process essentially based on trade-off analysis. It is impossible to obtain a solution that is the best for all considered objectives. In comprehending the mechanics of multi-objective optimisation in facilitating trade-off analysis, it becomes imperative to understand several foundational definitions. As interpreted by Chang [12], these definitions serve as crucial conceptual foundations within multi-objective optimisation frameworks.

Definition 1 Nondominated and Dominated. A Pareto optimal point has no other point that improves at least one objective without detriment to another; that is, it is non-dominated, vice versa.

Definition 2 Pareto front. A set of solutions that are non-dominated to each other but are superior to the rest of solutions in the search space.

**Fig. 2:**
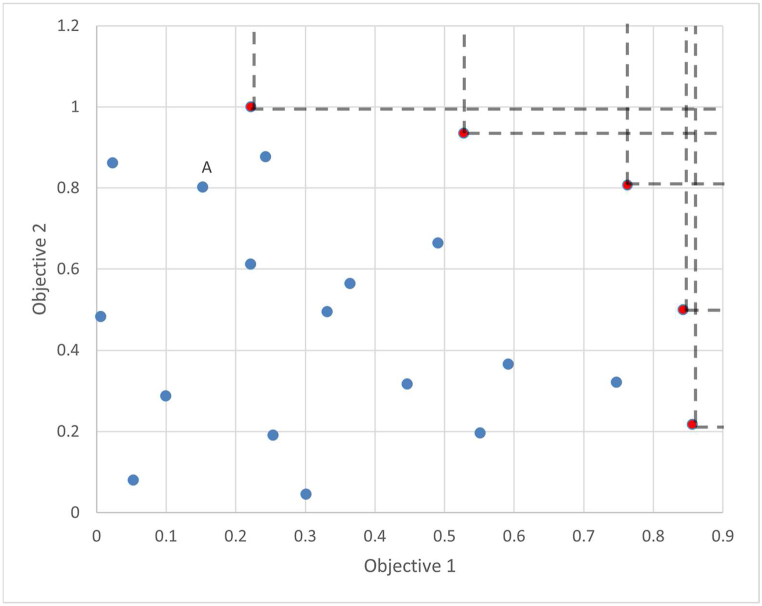
Random data set for definition illustration.

By elucidating these fundamental concepts, researchers gain a general understanding of the operational mechanisms governing trade-off analysis within the context of multi-objective optimisation. If a random data set is considered with two objectives aiming at maximization a plotted may be created as shown in Fig. 2.

As depicted in the above figure, the red dots are the set of non-dominated points called pareto front, each representing a unique trade-off between competing objectives. The Pareto front are deemed Pareto optimal (combination of countermeasures), as they offer the best achievable compromises across the objectives under consideration. Conversely, points located below or within the Pareto front as shown as blue dots are considered dominated, as they can be surpassed by other solutions in at least one objective without any corresponding improvement in other objectives.

### 3.2 Research methods

#### 3.2.1 Stage 1: multi-objective optimisation

The first stage involves generating a Pareto optimal set of solutions using the Non-Dominated Sorting Genetic Algorithm II (NSGA II) [25], implemented in R Studio. NSGA II is chosen for its ability to provide a better spread of solutions and convergence compared to other multi-objective evolutionary algorithms.

**Fig. 3:**
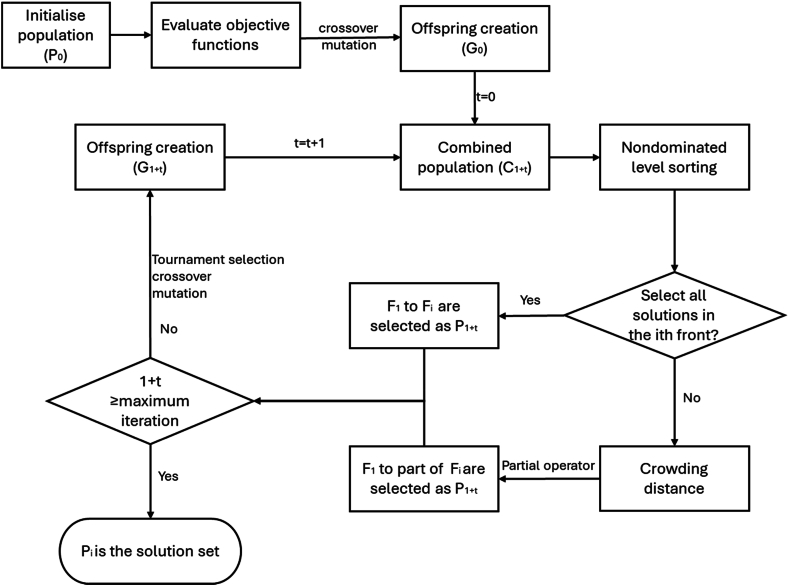
Flow chart of NSGA II.

It consists of two main steps: nondominated levels sorting and diversity preservation to search optimal solutions. The overall procedure of NSGA II is illustrated in Fig. 3 and demonstrated as followed. First, a random parent population of combinations of countermeasures (P_0_) of size N is initialised and evaluated using the objective functions to rank their front tier level. Offspring (G_0_) of size N is created by elitism election recombination, and mutation operators. The loop will begin once the first initial generation ends. The combined population of C_1_ is composed of P_0_ and G_0_ with size of 2 N. Nondominated levels sorting is used to rank the front tier level (F_i_) and search best solutions in the combined population of C_1_. Next, in order to identify the new population (P_2_) with size of N, which is composed of F_1_ to F_i_, crowding distance is calculated for each solution in ith front tier level to guarantee a good spread of solutions, where a partial operator was identified for binary tournament selection. Between two solutions, solution with a lower rank of front tier level is preferred; solution in a less crowded region if preferred if they are ranked as the same front tier level. The new population (P_1_) of size N is now used for selection, crossover, and mutation to create an offspring (G_1_) to make up the new combined population of C_2_. The process will iterate until the maximum generation is achieved. It should be noted that more than one optimal solution may be found.

#### 3.2.2 Stage 2: association rule mining

In the second stage, association rule mining is applied to weigh the optimal solutions through an aggregation method. This process uses a rule-based model with "if …, then … " decision rules.

Once nondominated solutions are searched out, it is necessary to identify the most suitable solution based on different weight criteria. Some studies [9,11] tried to employ multi criteria and interactive method to select the best solution based on experts’ preference and predefined criteria. Such methods are subjective resulting in bias and neglect of the importance of historical data. Thus, association rule mining is introduced to provide data-driven evidence in the context of road engineering.

Association rule mining is a widely used data mining method (Agrawal et al., 1994) to extract hidden information for road safety, which reflects the overall safety performance and potential problems of road network in the form of “if … then …”. There is a crucial parameter named lift indicating the robustness of association between antecedent (e.g. road type) and consequent (e.g. fatalities) in a rule [26]. It quantifies the relationship between the observed support and the support that would be anticipated under the assumption of independence between the left-hand side (LHS) and right-hand side (RHS). Therefore, this study employs lift values as indicators to measure the strength of associations contributing to KSI crashes.

The value of lift implies how strong association of a rule, where a value of 1 indicates the items in rules are independent, a value of over 1 indicates items are more likely to occur than if items were independent to each other. A higher lift value in a rule signifies a greater likelihood of resulting in killed or serious injury outcomes. Therefore, road attributes data at all sites and road sections for proposed road improvements are required to carry out lift weight aggregation.

Given that the performance of investment plan is based on overall countermeasure effectiveness, resulting in various characteristics within the same road attribute category, the traits with higher lift values are used for lift aggregation. Ultimately, the lift aggregation of countermeasure is calculated by summing the lifts of all associated road attributes. Similarly, the lift aggregation of combinations is the sum of lift of all selected countermeasures, which stands for the risk level of roads where these selected countermeasures are required to enhance road safety.

$$Lift\;aggregation\;of\;combination=\sum_{i=1}^{i}k_{i}\;\left(\;\sum_{n=1}^{n}l_{n,i}\right)$$

Where n indicates the nth road attributes associoated to fatal crash, i denotes the ith countermeasure proposed for road investment plan, $l_{n,i}$ is the lift value of the rule specified the strongness of association between nth attribute and KSI outcome for ith countermeasure option, and $k_{i}$ is a binary parameter denoting that $k_{i}$ = 1, if the ith countermeasure is selected in the combinations, otherwise, $k_{i}$ = 0.

### 3.3 Modelling

#### 3.3.1 Multi-objective identification

The optimisation model is structured to address multiple objectives and the objectives are considered from various perspectives. Benefit-cost analysis (BCA) and cost-effectiveness analysis (CEA) stand as two fundamental approaches for evaluating the efficacy of countermeasures. Within the iRAP methodology, the benefit-cost ratio (BCR) serves as a key metric for assessing countermeasures in economic terms. This involves computing the present value of safety benefits, considering the prevention of fatalities and serious injuries alongside the economic valuation of human life and severe injury. The costs associated with countermeasures are derived from construction expenses and data on service life. While BCR effectively quantifies the monetary implications of each countermeasure, decision-makers, operating under budgetary constraints, often aim to maximize the net present value of benefits, where the first objective is determined.

Behnood & Pino [27] proposed an optimisation framework grounded in cost-effectiveness analysis. Their procedure seeks to minimise the costs of countermeasures while simultaneously maximizing the reduction in the number of crashes. A critical aspect of this approach lies in understanding the resource allocation required for each additional unit of prevented fatalities and serious injuries. Accordingly, the second objective is identified as the minimization of the cost-effectiveness ratio (CER), which quantifies the resources expended per additional unit of prevented fatalities and serious injuries.

From the economical perspective, this model has chosen the service life as the third objective. Service life is a sensible measure in the way that it indicates the duration of the countermeasure after the implementation. Thus, the longer the service life, the more economical benefits (larger net present values) the countermeasures would yield for the same cost of implementation [9]. In addition, according to the study of Elvik [28] and Byaruhanga & Evdorides [29] the service life could affect the selection of countermeasures. In instances where the budget is sufficiently robust, prioritizing countermeasures characterized by long service life becomes instrumental in optimising economic advantages and ensuring enduring safety benefits. This strategic emphasis on countermeasures with continued effectiveness contributes to a reduction in treatment recurrence at specific locations, thereby minimizing disruptions to the operational efficiency of the road network.

Therefore, three objectives optimisation model is formulated focusing on: 1) maximize the net present value of benefits (see Eq. (1)); and 2) minimise the cost per KSI saved; (see Eq. (2)); and 3) minimise the total count of countermeasures characterized by a short service life (SSL) (see Eq. (3)).

Equation 1 $$\mathrm{MAX}\;f_{1}=\sum_{i=1}^{n}\left(B_{i}k\right)$$

Equation 2 $$\mathrm{MAX}\;f_{2}=\sum_{i=1}^{n}\left(E_{i}k\right)$$

Equation 3 $$\mathrm{MIN}\;f_{3}=\sum_{i=1}^{n}k_{i,SL=short}$$

In these equations, n represents the total number of recommended improvement activities outlined in the iRAP safer road investment plan and k is a binary parameter indicating the choice of selecting where k = 1 if the countermeasure is selected, otherwise k = 0. The parameter i signifies the $i^{th}$ countermeasure proposed for road enhancement, where $B_{i}$ denotes the NPV of benefits by implementing the $i^{th}$ countermeasure, and $E_{i}$ indicates the cost per KSI saved after implementing the $i^{th}$ countermeasure. SL is an abbreviation for service life, representing the duration for which the countermeasure remains effective. The variable $k_{i,SL=short}$ signifies the decision to implement the $i^{th}$ countermeasure with short service life (SSL). (where k = 1 if it is selected and it has short service lift, otherwise k = 0), and the main constraint of the model is the total budget (B) given by:

Equation 4 $$\sum_{i=1}^{n}C_{i}k\leq B$$

Where, $C_{i}$ denotes the cost of $i^{th}$ countermeasure.

#### 3.3.2 NSGA-II parameters setting

In the context of using NSGA-II for optimising Pareto front solutions within R Studio, it is critical to determine appropriate parameters for the algorithm. A key initial consideration is the population size. According to empirical studies [30,31], a population size ranging from 30 to 100 is often sufficient to explore the search space effectively while maintaining computational efficiency. In this specific scenario, a population size of 50 is chosen, as it provides a balance that allows for robust exploration and enables decision makers to compare and select different countermeasure combinations.

The decision variables in this optimisation problem are binary, representing whether a countermeasure is selected or not. The fitness function for this scenario encompasses three objectives: maximizing the net present value of benefits, minimizing the cost per KSI saved, and minimizing the total number of countermeasures with a short service life (SSL).

The maximum iteration count is set to 1000, and the nBits parameter, which specifies the number of bits used in the binary encoding of the optimisation problem, corresponds to the number of proposed countermeasures in the iRAP investment plan for Utrecht.

The crossover rate, which determines how frequently the crossover operator is applied, is set to 0.8 by default. This rate strikes a balance by introducing new structures into the population at a rate that avoids the premature loss of high-performance structures while still promoting adequate exploration. Mutation, which increases population variability, is set to a default probability of 0.1. This low mutation rate prevents any single bit position from converging prematurely while avoiding the randomness associated with higher mutation rates [25].

#### 3.3.3 Association rule mining parameters setting

For holistic road safety improvement, prioritizing the reduction of KSI incidents is a fundamental goal in road safety. There were an estimated 1.19 million road traffic deaths in 2021; this corresponds to a rate of 15 road traffic deaths per 100000 population [32]. In addition, EURoad Safety Policy Framework 2021–2030 aims at halving the number of fatalities and serious injuries on European roads by 2030, as a milestone on the way to ‘Vision Zero’ (European Commission, 2019), supporting the importance of understanding of KSI incidents to improve overall public safety and well-being. According to this, the consequent of the rule is set as KSI, so that all the rules extracted are related to KSI crashes on road network.

Minimum support and confidence are essential constraints for extracting useful information from data ([33]). To capture 99 % of the data corresponding to a specific consequent, the support value of that consequent is first calculated. The minimum support value is then set to 1 % of this support value. For instance, in 2014, there were 2799 crash records in Utrecht, 478 of which were KSI (Killed or Seriously Injured) crashes, resulting in a support value of 0.171. Consequently, the minimum support value is set to 0.002. Correspondingly, specifying confidence is less critical in this study since the primary parameter used is the lift value. Therefore, the minimum confidence is set to match the minimum support value, which is very low, reflecting patterns with weak association and frequency in the crash database.

Additionally, the number of items in each rule is set to two: one being the consequent of KSI (Killed or Seriously Injured) and the other representing patterns in the crash database. This study assumes that all onsite attributes of road crashes can independently influence crash severity. Consequently, the lift aggregation method can be employed to assess the risk level of road locations or segments where road improvements are proposed by the iRAP investment plan.

### 3.4 Data collection

In this study, three different types of databases are considered. One is the national road crash registration (BRON), which is based in SWOV, the national scientific institute for road safety research in the Netherlands. The other two are safer roads investment plan (SRIP) and road attribute data stored in Vida web software embedded in iRAP methodology, which come from iRAP implementation in Utrecht, Netherlands.

The BRON database is a collection of all road traffic crashes in the Netherland. The database contains detailed information on crash circumstances, such as the year, day, date, road type, speed limit, mode of transport, and more. It serves as a crucial resource for road safety research, enabling the analysis of various factors related to roads, road users, vehicles, and traffic movements. In addition, the data is extensively used for guiding improvements in road safety infrastructure which is highly reliable as it records onsite crash situations, making it an indispensable source for identifying road infrastructure issues. For this study, crash data from 2014 were extracted to develop the proposed procedure.

SRIP are the results of iRAP embedded in the Vida application, a web online tool, which has been elaborated in the methodology of iRAP [2]. There are plenty of road related data required to develop SRIP in ViDA, which also includes a calibration of fatalities estimation. Brief process of SRIP development is provided consisting of 6 steps. Firstly, triggers, the prerequisite conditions to select countermeasures where iRAP has more than 300 triggers been defined, are tested for further consideration of investment plan. Secondly, the number of deaths and serious injuries prevented is calculated. Thirdly, benefit cost ratio (BCR) analysis is carried out for screening out countermeasures of which BCR is below the predefined threshold. Fourthly, minimum length, minimum spacing and hierarchy rules were considered by analysis team to define their preference for selecting countermeasures. Fifthly, the impact of all viable countermeasures is determined at the 100-m level and the combined deaths and serious injuries after all viable countermeasures have been applied is calculated for each crash type. Lastly, the total number of deaths and serious injures prevented for the individual countermeasure at that location is then adjusted to take account of multiple countermeasures that affect the same crash type. At the completion of the countermeasure selection process, a final economic analysis for all the countermeasures selected across all roads is undertaken and SRIP is produced. The SRIP data is highly detailed and structured, providing a thorough assessment of countermeasure effectiveness and economic viability based on road sections and location safety analysis. The example of data with performance of each countermeasure is summarised in Table 1.

**Table 1:** Example of safer road investment plan data.

Countermeasure	Length/Sites	FSIs saved	PV of safety benefit	Estimated Cost	Net monetary benefit	Cost per KSI save	life
Additional lane (2 + 1 road with barrier)	15.10 km	80	25660031	20655000	5005031	258188	medium
Bicycle Lane (off-road)	3.50 km	3	1122681	392156	730525	130719	medium
Central hatching	5.70 km	0.6	184481	34173	150308	56955	medium
Central median barrier (1 + 1)	34.80 km	41	13286137	6288200	6997937	153371	medium
Central median barrier (no duplication)	0.70 km	1	365701	95182	270519	95182	medium
Centreline rumble strip/flexi-post	1.80 km	0.3	108423	19512	88911	65040	low
Clear roadside hazards (bike lane)	1.20 km	0.5	160633	216000	−55367	432000	low
Clear roadside hazards - driver side	0.10 km	0.1	34232	20000	14232	200000	low
Delineation and signing (intersection)	8 sites	0.5	149378	88582	60796	177164	low
Duplication with median barrier	1.20 km	26	8323159	6480000	1843159	249231	high
Footpath provision driver side (>3 m from road)	25.40 km	28	9060036	2922040	6137996	104359	low
Footpath provision driver side (adjacent to road)	27.90 km	35	11319681	4388280	6931401	125379	low
Footpath provision driver side (informal path >1 m)	4.70 km	1	307139	114747	192392	114747	low
Footpath provision passenger side (>3 m from road)	26.80 km	29	9267974	3085368	6182606	106392	low
Footpath provision passenger side (adjacent to road)	44.10 km	52	16843531	6939120	9904411	133445	low
Footpath provision passenger side (informal path >1 m)	5.80 km	1	406768	141451	265317	141451	low
Improve curve delineation	0.40 km	0.5	148419	7460	140959	14920	low
Improve Delineation	45.70 km	13	4190892	847446	3343446	65188	low
Lane widening (>0.5 m)	0.10 km	0.6	186272	152529	33743	254215	low
Lane widening (up to 0.5 m)	1.00 km	2	684333	656756	27577	328378	low
Overtaking lane	0.30 km	1	339394	405000	−65606	405000	medium
Parking improvements	1.50 km	0.3	99666	18900	80766	63000	low
Pedestrian fencing	27.20 km	9	2976955	179606	2797349	19956	medium
Protected turn lane (unsignalised 3 leg)	54 sites	74	23918473	7210571	16707902	97440	low
Protected turn lane (unsignalised 4 leg)	3 sites	14	4375323	535399	3839924	38243	low
Protected turn provision at existing signalised site (4-leg)	1 sites	0.6	204497	237955	−33458	396592	low
…							

Road attribute data, 78 attributes in total coded and stored in ViDA, are collected during road inspection by operating road survey and road coding. Road survey requires a detailed of geo-referenced image data of a road network and road coding records road attribute categories using survey images. The road attribute data is precise and systematically recorded, ensuring a comprehensive representation of road conditions for further development of SRIP, where attribute data are extracted based on locations or road segments of each proposed countermeasure. Compared road crash data in Netherland and ViDA embedded road attribute data, four distinct representative attributes are identified, which are recorded in both databases with expected significance to road infrastructure safety improvement. The road attributes data related to countermeasures is summarised in Table 2.

**Table 2:** Example of road attribute data related to countermeasures.

Countermeasure	Length/Sites	Urban/rural town or rural/open area	Carriageway label	Street lighting	Speed limit (km/h)	Junction
Additional lane (2 + 1 road with barrier)	15.10 km	rural	Single car'way	partly	80	not
Bicycle Lane (off-road)	3.50 km	both	both	present	50/80	not
Central hatching	5.70 km	urban	Single car'way	present	50/60/80	not
Central median barrier (1 + 1)	34.80 km	rural	Single car'way	partly	60/70/80	not
Central median barrier (no duplication)	0.70 km	rural	Single car'way	present	70/80	not
Centreline rumble strip/flexi-post	1.80 km	rural	Single car'way	present	50/60/70/80	not
Clear roadside hazards (bike lane)	1.20 km	both	Single car'way	present	60/80	not
Clear roadside hazards - driver side	0.10 km	urban	Single car'way	present	50	not
Delineation and signing (intersection)	8 sites	both	Single car'way	present	50/60/80	at
Duplication with median barrier	1.20 km	rural	Single car'way	present	80	not
Footpath provision driver side (>3 m from road)	25.40 km	rural	Single car'way	partly	50/60/80	not
Footpath provision driver side (adjacent to road)	27.90 km	rural	both	partly	50/60/80	not
Footpath provision driver side (informal path >1 m)	4.70 km	rural	Single car'way	partly	50/60	not
Footpath provision passenger side (>3 m from road)	26.80 km	rural	both	partly	50/60/70/80	not
Footpath provision passenger side (adjacent to road)	44.10 km	rural	both	partly	50/60/70/80/100	not
Footpath provision passenger side (informal path >1 m)	5.80 km	rural	both	partly	50/60/70	not
Improve curve delineation	0.40 km	rural	both	present	80	not
Improve Delineation	45.70 km	both	both	partly	50/60/80/100	not
Lane widening (>0.5 m)	0.10 km	rural	Single car'way	present	80	not
Lane widening (up to 0.5 m)	1.00 km	rural	Single car'way	present	70/80	not
Overtaking lane	0.30 km	rural	Single car'way	present	70/80	not
Parking improvements	1.50 km	both	Single car'way	partly	60/50/80	not
Pedestrian fencing	27.20 km	both	both	partly	50/60/70/80/100	not
Protected turn lane (unsignalised 3 leg)	54 sites	rural	Single car'way	partly	50/60/80	at
Protected turn lane (unsignalised 4 leg)	3 sites	rural	Single car'way	present	80	at
Protected turn provision at existing signalised site (4-leg)	1 sites	rural	Dual car'way	present	80	at
…						

## 4 Result

### 4.1 Result of optimisation model

Budget constraint used in this study were identifies from the user-defined investment plan determined by the royal Dutch tourist association (ANWB). They ultimately chose 30 countermeasures out of 47 countermeasures recommanded by iRAP to improve the provincial road in Utrecht, which estimated saving 777 fatalities and serious injuries, 250 million euro, with benefit cost ratio of 2.2 by costing 113 million euro. Therefore, the budegt constraint was set to 113 million euro to validate the advancements of the methodology proposed in this study.

**Fig. 4:**
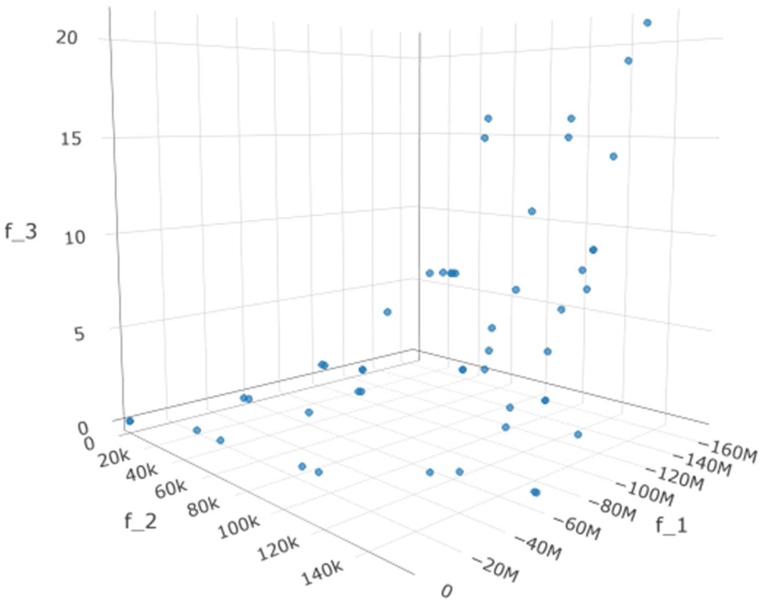
Visualization of Pareto front.

After eliminating duplicates and solutions exceeding the budget constraint, 45 optimal solutions emerged. These solutions were assessed based on their objective values, as detailed in Table 3, and the findings were visually presented in the accompanying Fig. 4. The net present value of benefits, as determined by ANWB, amounts to 135.7 million euros. Among the 45 solutions, 8 of them (designated as solutions 1, 12, 20, 23, 24, 25, 28, and 43) outperform ANWB's investment decision, specifically in maximizing the net present value of benefits. It costs 147,281 euros to save each KSI occupant in their investment plan, with only two solutions (designated as solutions 6 and 8) performing less favourably compared to ANWB's decision, while the majority of solutions prove to be more cost-effective. According to investment strategy determined by ANWB, which involves the selection of 19 countermeasures with short service life, it is noted that over half of the improvements necessitate reconstruction or rebuilding within a period of one to five years. This would result in periodic efforts and potentially disrupt the standard functioning of the road network significantly. Among the 45 solutions evaluated, only two investment combinations opt for at least 19 countermeasures with short service lives, while the remaining alternatives exhibit superior performance with respect to the third specified objective.

**Table 3:**
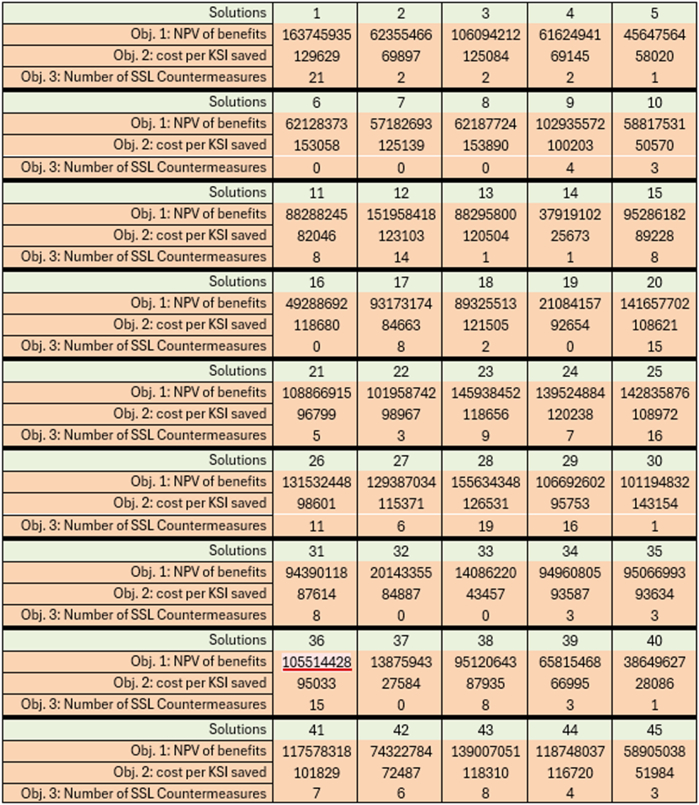
Objective value of optimal solutions.

### 4.2 Result of integrated methodology

Once the optimal solutions are searched, all alternative combinations are employed to weight assignment process. As mentioned in methodology, the lift value is used as a weight metric in the form of rule to indicate the association between road attributes to KSI result, where association rule mining is applied to explore their association. Rules were extracted and demonstrated in Table 4.

**Table 4:** Decision rules associated to KSI crash.

LHS	RHS	support	confidence	lift
Inside outside built-up areas = Urban area	KSI	0.005359057	0.6	3.513389
Inside outside built-up areas = Rural area	KSI	0.005716327	0.484848485	2.839102
Speed limit road = 60 km/h	KSI	0.011432655	0.32	1.873808
Situation of road = Bend	KSI	0.016434441	0.238341969	1.395647
Situation of road = Intersection - 3 legs	KSI	0.019649875	0.224489796	1.314533
Road surface = Concrete	KSI	0.00785995	0.211538462	1.238695
Road surface = Porous asphalt	KSI	0.034297964	0.210065646	1.230071
Speed limit of road = 100 km/h	KSI	0.016791711	0.209821429	1.228641
Speed limit of road = 120 km/h	KSI	0.010360843	0.204225352	1.195872
Situation of road = Intersection - 4 legs	KSI	0.03965702	0.198214286	1.160673
Street lighting = Not burning	KSI	0.123258307	0.18935236	1.108781

According to the attributes derived from ViDA database and the result of crash databases rule mining analysis, four distinct risk attributes are used for lift aggregation method involving the road area, speed limit, situation of road, and street lightning. To highlight the urgency and significance of countermeasures recommended by ViDA across diverse locations and sections, the level of risk associated with roads will be signified by greater lifts of attributes among varying road segments. For instance, in the scenario where the construction of refuge islands is recommended for both three-leg and four-leg intersections, the lift value observed for the three-leg intersection will serve as a measure for urgency due to its comparatively higher lift. The top ten solutions ranked by lift aggregation with their objective value are demonstrated in Table 5.

**Table 5:** Ranking of investment plan.

Investment alternatives	Objective 1 (Maximize)	Objective 2 (Minimise)	Objective 3 (Minimise)	Lift aggregation	Ranking
1	163745935	129628.5	21	163.85	1
29	155634348	126531.3	19	145.06	2
26	142835876	108971.9	16	134.27	3
13	151958418	123103	14	132.94	4
30	106692602	95752.54	16	128.32	5
21	141657702	108620.5	15	119.24	6
37	105514428	95032.68	15	113.29	7
25	139524884	120238	7	96.42	8
24	145938452	118656	9	93.05	9
16	95286182	89227.85	8	89.47	10
Utrecht Plan	135739970	135739970	19	–	–

## 5 Discussion

### 5.1 Model performance of case study

It is impossible to obtain a solution that is the best for all considered objectives, whereas it is imperative to discover the pareto front tier where a set of optimal solutions could be identified for decision makers. This approach enables an evidence-based assessment of prioritization concerning differential risk levels identified across distinct road infrastructure configurations, thereby informing the allocation of resources and interventions in road safety management. Contrasted with the investment plan outlined in the Utrecht report, which involved the selection of 33 countermeasures within a budgetary allocation of 114378840 euros, their strategy selected for 19 countermeasures characterized by a limited operational lifespan of fewer than 5 years. This selection was estimated to generate a net present value of benefits amounting to 135739970 euros, with an associated cost of 147282 euros per KSI saved. However, our model diverged in its selection.

The top 10 alternatives are ranked by lift aggregation. In other word, these alternatives concern road locations and sections with high risky road attributes that are very likely to cause KSI crash. Each objective will be discussed separately to validate the advancement of our methodology. With regards to the objective 1 (Obj.1), aimed at maximizing the NPV of benefits, it is noteworthy that alternative 1 shows a visible enhancement of about 20 % when compared with the NPV estimation described in the Utrecht report. This improvement implies the advanced ability of our proposed methodology in searching the best solution for maximizing monetary gains.

As for objective 2 (Obj.2), which strives to minimise the cost per KSI saved, alternative 16 emerges as a standout performer, with an average cost of 89,227.85 euros per KSI saved. This represents a significant improvement from the Utrecht plan, where the cost per KSI saved is approximately 147,281.5 euros, thereby signifying a substantial 39 % reduction in anticipated expenditures. This marked reduction underscores a valuable advancement by applying the proposed method in cost-effectiveness measures.

Regarding the objective 3 (Obj.3), trying to minimise the number of countermeasures with a short lifespan of less than 5 years selected, the Utrecht report identified a total of 19 such countermeasures. In contrast, Alternative 25 selected only seven short-lived road improvement interventions. This plan is anticipated to result in reduced frequency of construction activities and decreased disruption to the operation of the road network. This discrepancy also highlights the capacity to identify optimal solutions in quantitative terms. In addition, the proposed methodology facilitates the proficiency in simultaneously optimising various objectives across different metrics as it is a critical consideration given that real-world objectives typically extend beyond financial concerns to encompass diverse units of measurement.

**Fig. 5:**
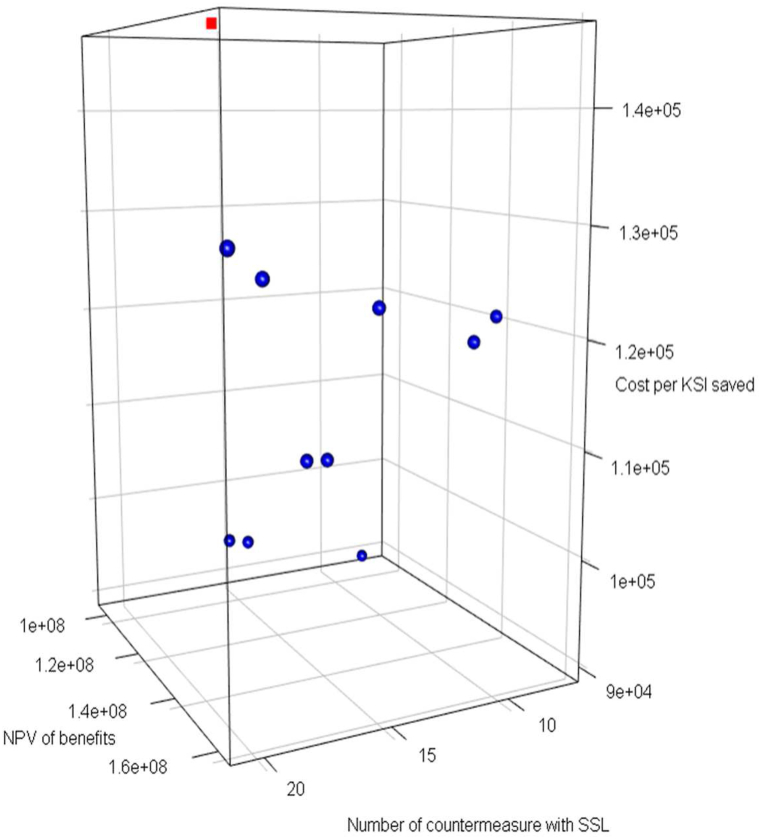
3D visualization of top 10 investment plan.

Despite the presence of trade-offs among the three objectives, the model identifies top 10 optimal countermeasure combinations based on lift aggregation. Note that we aim to maximize NPV of benefits (Obj.1).while minimise Cost per KSI saved (Obj.2) and number of countermeasure with SSL (Obj.3).The 3D visualization depicts the 10 alternatives as blue circles, comparing with the investment plan of Utrecht represented by red squares as show in Fig. 5. Intriguingly, all solutions outperform in Obj.2, which suggests that these investment plans are particularly effective in reducing costs associated with preventing KSIs. Half of these solutions show superior performance across all three objectives compared to the investment plan formulated by ANWB. This indicates that these solutions not only excel in cost-effectiveness but also in achieving other objectives related to safety.

**Fig. 6:**
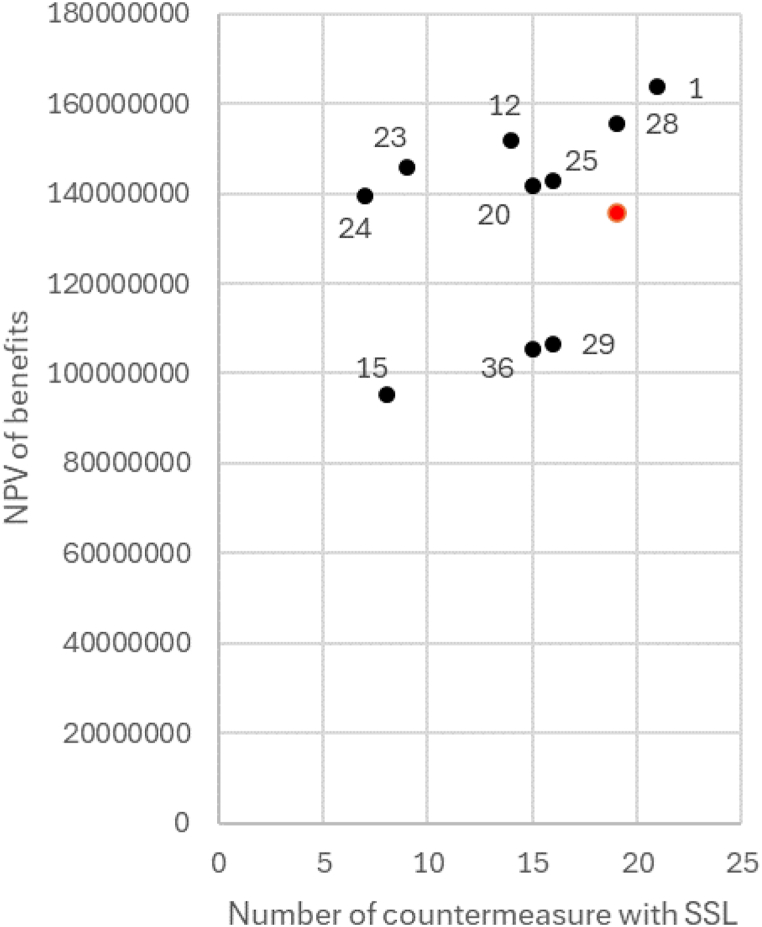
Two objectives analysis.

Due to their exceptional performance regarding the second objective, a 2D visualization is presented for a clearer understanding of the performance and compromises of 10 investment plans considering the first and third objectives as illustrated in Fig. 6. It interprets the comparative performance of various solutions in terms of objective 1 (NPV of benefits) and objective 3 (Number of countermeasures with SSL). Notably, alternatives 15, 29, and 36 exhibit a strategic inclination towards reducing the number of Short Service Lift (SSL) countermeasures, with sacrifice of the Net Present Value (NPV) of benefits. Conversely, combinations 1 and 28 prioritize the maximization of NPV of benefits, even employing an equal or greater number of SSL countermeasures. Remarkably, the methodology employed in this study has found five solutions (12, 20, 23, 24, and 25) which simultaneously optimise three objectives to varying extents.

This optimisation method is contributory in realizing substantial value within resource constraints, featuring the multiple considerations and diverse measurement metrics involved. This highlights the value of the proposed methodology in achieving a balance between competing priorities and underscores its potential to outperform conventional planning strategies in optimising outcomes across multiple dimensions.

In recent years, there has been a prevalence of single-objective optimisation methods in the road context, each focusing on different aspects. These methods commonly revolve around optimising specific performance index Chowdhury et al. [7], minimizing the number of crashes and time delays [6], or considering the service life of countermeasures [29]. In addition, some multi-objective optimisation models have been developed, all rely heavily on expert input, often involving subjective evaluations of stakeholder needs and preferences [8], two-level optimisation processes by classifying decisions and supporting objectives [9], or the formulation of decision rules by experts [11].

In contrast, the proposed two-stage model not only considers diverse objectives, even when they differ in units, but also provides data-driven evidence of road context characteristics with minimal expert efforts to allocate resource. The contribution of this study is twofold. As demonstrated by the prototype developed in this study, the optimisation model effectively searches the Pareto frontier across various objectives within the constrained budget. Specifically, it underscores the practical applicability of the model in real-world decision-making scenarios and provides empirical evidence of the effectiveness of the non-dominant sorting genetic algorithm in identifying the Pareto frontier. Furthermore, this study highlights the potential of data-driven preferences to guide resource allocation and offers a framework for integrating data-driven insights with less expert efforts to enhance decision-making in complex road management scenarios.

This model was developed by leveraging the outcomes of an investment plan derived from the iRAP methodology. This approach empowers local authorities to devise user defined investment plans tailored to the specific characteristics of their local road network environment. While not advocating for complete elimination of expert control, this model empowers experts with greater insights from natural data pertaining to diverse road contexts, thereby reducing the pressure associated with searching for optimal solutions.

### 5.2 Challenges and limitations

The proposed methodology, while demonstrating substantial potential in optimising resource allocation and enhancing road safety, presents several challenges and limitations that must be acknowledged.

A significant challenge lies in the extensive data requirements of the methodology. High-quality and comprehensive historical crash data, alongside detailed road attribute data, are essential for analysis. However, acquiring such data can be problematic, especially in regions with limited data collection infrastructure or inconsistent data recording practices. The variability in data quality and completeness can adversely affect the reliability of the results.

The study is conducted based on the overall performance of specific countermeasures derived from iRAP investment plan. However, different road sections or junctions may exhibit varied road attributes within the same countermeasure category, leading to a potential oversimplification in the analysis. Enhanced specificity could be attained by formulating investment plans based on homogeneous sections and junctions, each characterized by distinct countermeasure performance metrics. This would allow for more precise and effective countermeasure selection, tailored to the unique characteristics of specific road segments.

The current approach relies on the lift value derived from association rule mining to determine the weight of risk levels. This method assumes that identified road attributes, or risk factors, independently signify the urgency for countermeasures. However, this assumption overlooks potential interactive relationships among these attributes. Consequently, there is a risk of overestimating the risk levels associated with specific road sections or junctions, potentially leading to an exaggerated sense of urgency and misallocation of resources. A more detailed risk assessment approach that accounts for the interdependencies among road attributes would enhance the accuracy and effectiveness of the proposed methodology.

In conclusion, although the proposed methodology shows significant promise in optimising resource allocation and improving road safety outcomes, several challenges and limitations need to be addressed. The extensive data requirements, the need for a correlated risk identification are critical areas that require further attention. Future research should consider incorporating comprehensive datasets from different regions and contexts to thoroughly evaluate the performance of proposed model. Addressing these limitations will enhance the reliability and practicality of the methodology, making it a more robust tool for road safety management.

## 6 Conclusion

This study highlights the originality and importance of its methodology in addressing the complexities of multi-objective optimisation for road safety strategy development. By overcoming challenges associated with simultaneous optimisation of multiple objectives, this approach offers a novel solution that promises to improve decision-making processes and optimise resource allocation in road infrastructure management. Additionally, the study emphasizes the practical implications of the methodology by providing a systematic framework that integrates diverse objectives and decision rule mining. This equips road agencies and stakeholders with a valuable tool for more efficient resource allocation and road safety improvement, requiring less human intervention.

However, it is crucial to acknowledge the challenges associated with the quantity and quality of data, particularly in the context of developing countries. The performance of our methodology relies heavily on the availability and reliability of data, highlighting the need for ongoing efforts to improve data collection processes and enhance data quality assurance measures. Furthermore, while the methodology effectively addresses the complexities of multi-objective optimisation, there remains a need for refinement of identification of risk level considering interactive effects of multiple road attributes. Future research should focus on formulation of investment plans centred on homogeneous road sections and junctions and developing more sophisticated models that can capture these interactions more comprehensively, thereby enhancing the effectiveness of the methodology. In conclusion, while the methodology represents a significant advancement in the field of road safety strategy development, there is still much work to be done to fully realize its potential.

## Data availability statement

Sharing research data enables other researchers to evaluate findings, build upon previous work, and enhance trust in published articles. Authors make their data as publicly accessible as reasonably possible. Below is a summary of the data availability for this study, which will be published alongside the article.

The data associated with this study has been deposited in a publicly accessible repository. Data utilized in this study were obtained from the National Road Crash Registration (BRON) database, managed by SWOV, the Safer Roads Investment Plan (SRIP) data from the ViDA application based on the iRAP methodology, and the road attribute data also stored in ViDA. Reproduction of this data is authorized, provided the source is acknowledged. Interested parties can access the data through the following link:

SWOV. (2014). National Road Crash Registration (BRON) database. SWOV, The Netherlands. Available at: https://swov.nl/en/data/crashes.

iRAP. (2014). Safer Roads Investment Plan (SRIP) data from ViDA application. iRAP, Utrecht, The Netherlands. Available at: https://vida.irap.org/en-gb/dashboard.

iRAP. (2014). Road attribute data from ViDA application. iRAP, Utrecht, The Netherlands. Available at: https://vida.irap.org/en-gb/dashboard.

## CRediT authorship contribution statement

**Bosong Jiao:** Writing – review & editing, Writing – original draft, Visualization, Validation, Software, Methodology, Investigation, Formal analysis, Data curation, Conceptualization. **Harry Evdorides:** Writing – review & editing, Supervision.

## Declaration of competing interest

The authors declare that they have no known competing financial interests or personal relationships that could have appeared to influence the work reported in this paper.
